# δEF1 Down-Regulates ER-α Expression and Confers Tamoxifen Resistance in Breast Cancer

**DOI:** 10.1371/journal.pone.0052380

**Published:** 2012-12-21

**Authors:** Shaocong Guo, Yaqing Li, Qi Tong, Feng Gu, Tianhui Zhu, Li Fu, Shuang Yang

**Affiliations:** 1 Medical College of Nankai University, Tianjin, China; 2 Tianjin Medical University Cancer Institute and Hospital, Tianjin, China; Dartmouth, United States of America

## Abstract

Resistance to tamoxifen therapy represents a major barrier to the successful treatment of breast cancer, where a loss of or reduced ER-α level is considered a primary mechanism. Understanding how ER-α expression is regulated would provide insights into new intervention points to overcome tamoxifen resistance. In this study, we report that the expression of δEF1 is up-regulated by 17β-estradiol (E2) in MCF-7 cells in an ER-α-dependent manner, through either PI3K or NF-κB pathway. Ectopic expression of δEF1 in turn repressed ER-α transcription by binding to the E_2_-box on the ER-α promoter. At the tissue level of breast cancer, there is a strong and inverse correlation between the expression levels of δEF1 and ER-α. In MCF-7 cells, an elevated expression of δEF1 made the cells less sensitive to tamoxifen treatment, whereas overexpression of ER-α compromised the effects of δEF1 and restored the sensitivity. Also, depletion of δEF1 by RNA interference in MDA-MB-231 cells restored the expression of ER-α and tamoxifen sensitivity. In conclusion, we have identified an important role of δEF1 in the development of tamoxifen resistance in breast cancer. Inhibiting δEF1 to restore ER-α expression might represent a potential therapeutic strategy for overcoming endocrine resistance in breast cancer.

## Introduction

Breast cancer is a classical model to study hormone-dependent tumors. Estrogen plays a major role in the development and progression of breast cancer. Nearly 70% of breast cancer expresses estrogen (ER) and/or progesterone (PR) receptors, which is an ER-dependent gene product. Thus, targeting ER using SERMs (selective estrogen-receptor modulators) represents a reliable therapeutic modality for all stages of this disease. As the most potent SERM, tamoxifen has been used as a major adjuvant treatment for primary breast cancer. However, over 50% of ER-positive tumors that initially respond to tamoxifen therapy will eventually develop resistance, resulting in recurrence and progression of the cancer and the subsequent death of patients [1], [2]. Knowledge so far on the possible causes for the intrinsic and acquired resistance have been attributed to the pharmacological property of tamoxifen, alterations in the expression and function of ER, interactions of tumors with local microenvironment, and genetic alterations of tumor cells [3]–[6]. To date, no dominant molecular mechanism leading to the resistance has been identified.

δEF1 (δ-crystallin enhancer factor 1), a member of the zinc finger-homeodomain transcription factor family, modulates cell differentiation and tissue-specific cellular functions [7]–[16]. Expression of δEF1 is implicated in the differentiation of multiple cell lineages, including bone [9], [13], [14], smooth muscle [11], neural [12], and T-cells [15]. δEF1 is also a key regulator of malignant progression of various tumors, including breast [17]–[19], pancreatic [20], squamous [21], and uterine [22] tumors. In breast cancer cells, δEF1 functions as a switch between proliferation and differentiation and promotes a more malignant phenotype [23]–[27]. At the molecular level, Dillner *et al.* reported that δEF1 mediates the estrogen-activated transcription of the *ovalbumin* (*Ov*) gene in the chick oviduct [28], [29]. Built on our previous finding that δEF1 is inversely related with the functional cascade of estrogen/ER [25], we speculated that δEF1 may play a role in tamoxifen resistance in the breast cancer.

To address this issue, we treated MCF-7 cells with 17β-estradiol (E2), which up-regulated δEF1 expression. Inhibition of ER-α significantly decreased the E2-induced expression of δEF1, whereas the ectopic expression of δEF1 down-regulated ER-α, rendering the MCF-7 cells less sensitive to tamoxifen. Chromatin immunoprecipitation (ChIP) assays demonstrated that δEF1 represses the transcriptional of ER-α promoter activity by binding to its E_2_-box. Notably, in the breast cancer specimens, we found a strong inverse correlation between δEF1 and ER-α protein expression. Taken together, our data demonstrated a negative feedback loop in the estrogen/ER cascade which is δEF1-dependent and confers tamoxifen resistance in breast cancer.

## Materials and Methods

### Tissue Samples

Fresh breast cancer tissues of invasive ductal carcinoma were obtained from the Tissue Banking Facility that is jointly supported by Tianjin Medical University Cancer Institute and Hospital (TMUCIH) and National Foundation for Cancer Research (NFCR). Tissue samples from a total of 120 subjects were collected, among which 60 were ER/PR-positive. All patients had histologically confirmed breast cancer and were recruited by the same department. The study was approved by the institutional ethics committee.

### Plasmid Constructions

Generation of full-length δEF1-myc expression vectors was previously described [25]. Human ER-α (−3998/−1783) promoter was amplified by PCR from the genomic DNA of human blood cells. The promoter was cloned into the pGL3-promoter vector (Promega, WI, USA) using the forward primer, 5′-ACAGGTACCGTTAGGACAGGTAAGGTAATGGGTC-3′, and the reverse primer, 5′-ATACTCGAGCGAGGTTAGAGGCGACGC-3′. The mutagenesis of the E_2_-box element on the ER-α promoter was performed using the Quick Change Site-Directed Mutagenesis Kit (Stratagene, CA, USA) with the forward primer, 5′-caatctttacccttctt**catctg**agagagccagtaagtc-3′, and the reverse primer, 5′-gacttactggctctct**cagatg**aagaagggtaaagattg-3′.

### Cell Culture and Transfection

Human breast cancer cell lines MCF-7 and MDA-MB-231 were maintained in DMEM-hi glucose medium (Invitrogen, CA, USA) that was supplemented with 10% FBS (Invitrogen, CA, USA), penicillin, and streptomycin. The cells were transfected using TurboFect™ Transfection Reagent (Fermentas, MI, USA) according to the manufacturer’s protocols.

Transient transfection with δEF1 expression plasmid was performed using the TurboFect™ Transfection Reagent on MCF-7 or MDA-MB-231 cells. Blasticidin- or G418-resistent clones were isolated over a period of 3–4 weeks. The overexpression or knockdown of δEF1 was confirmed by Western Blot.

### RNA Extraction and Quantitative PCR (Q-PCR)

Using the TRIzol Reagent (Invitrogen, CA, USA), total RNA was extracted from MCF-7 cells that were either treated with E2 or transfected with the δEF1 expression plasmid. Total RNA (0.5 µg) from each sample was used for first-strand cDNA synthesis using M-MLV Reverse Transcriptase (Promega, WI, USA). Specific products of human δEF1 and ER-α were amplified by Q-PCR using the following primers: δEF1, 5′-ATGTGGCTCAGTTTGTCCTC-3′ (forward) and 5′-AGCAAGATTTCCTCCAGGTC-3′ (reverse) and ER-α, 5′-GAAGAGGAGGGAGAATGTTG-3′ (forward) and 5′-ACTGAAGGGTCTGGTAGGAT-3′ (reverse). Verification of the expression levels of the genes was performed by Q-PCR using EvaGreen (Biotium, CA, USA). GAPDH was used as an internal control.

### Preparation of Short Hairpin RNAs (shRNAs)

The shRNA target sequences for human ER-α and δEF1 were 5′-ACAGGAGGAAGAGCTGCCA-3′ and 5′-TGATCAGCCTCAATCTGCA-3′, as previously reported [27], [30]. Sense and antisense oligonucleotides with internal loops were synthesized (TaKaRa, Shiga, Japan). These were annealed and ligated into the BamHI and HindIII sites of pSilencer 4.1-CMVneo (Ambion, CA, USA) to construct the ER-α-specific shRNA expression plasmid (shERα) and δEF1-specific shRNA expression plasmid (shδEF1) according to the manufacturer’s protocols. pSilencer 4.1-CMVneo expressing scrambled shRNAs (Ambion, CA, USA) were used as controls.

### Western Blot and Antibodies

Preparation of total cell extracts and Western Blot with appropriate antibodies were performed as described [9]. The following antibodies (Abs) were used at 1∶1000 dilutions: a rabbit polyclonal Ab against δEF1 (sc-10572, Santa Cruz, CA, USA), rabbit polyclonal Ab against ER-α (sc-542, Santa Cruz, CA, USA), and mouse monoclonal Ab against Actin (A-4700, Sigma, MO, USA).

### Immunofluorescence Microscopy

MCF-7 cells were grown on glass chamber slides and treated with inhibitors of the indicated signaling pathways (PI-103 for PI3K, PKI for PKA, and BAY11-7082 for NF-κB pathways) in the presence or absence of E2. The cells were fixed using 4% paraformaldehyde in PBS for 30 min and permeabilized in 0.1% Triton X-100 for 30 min and then blocked with 0.5% BSA in PBS for 30 min at RT. After washing with PBS, the cells were incubated with the anti-δEF1 antibody (sc-10572, Santa Cruz, CA, USA) for 2 h at 37°C. After washing with PBST, the cells were incubated with the appropriate fluorescein isothiocyanate-conjugated secondary antibody and stained with 4′,6-diamidino-2-phenylindole (DAPI) (Roche, Basel, Switzerland). Images were visualized and captured using an Olympus microscope.

### Luciferase Assay

MCF-7 cells were co-transfected with either the wild-type or mutant ER-α promoter constructs and the δEF1 expression plasmid in 24-well plates. Cell lysates were prepared and luciferase activities were measured using the Dual-Luciferase Reporter Assay System (Promega, WI, USA) according to the manufacturer’s protocols. The luciferase activities were normalized using the Renilla luciferase activities.

### ChIP Assays

ChIP assays were performed following a protocol described previously [25]. Briefly, MDA-MB-231 cells were transfected with the δEF1 expression plasmid or empty vector control. The cell lysates were prepared using a ChIP assay kit (Upstate, NY, USA) following the manufacturer’s guidelines. Chromatin solutions were precipitated overnight with agitation at 4°C using 10 µL of rabbit polyclonal Ab against δEF1 (sc-10572, Santa Cruz, CA, USA) or anti-rabbit normal IgG (sc-2345, Santa Cruz, CA, USA). The amounts of each specific DNA fragment in the immunoprecipitates were determined by PCR or Q-PCR reactions. The fragment of human ER-α promoter containing the E_2_-box site was amplified using the forward primer, 5′-CACCAAGTGATTCCAA-3′, and the reverse primer, 5′-GGGATATTGGAGCAGC-3′.

### Immunohistochemical Analysis

Immunohistochemical analysis was performed on paraffin-embedded sections using the Envision Kit (Dako, Glostrup, Denmark) following the manufacturer’s protocols. Sections were boiled in retrieval solutions to expose the antigens. Polyclonal anti-δEF1 (sc-10572, Santa Cruz, CA, USA), polyclonal anti-ER-α (sc-542, Santa Cruz, CA, USA), and control primary antibodies were applied to the sections at a dilution of 1∶100. The section-affixed slides were counterstained with hematoxylin, dehydrated, and mounted. The immunostaining results were evaluated independently by two pathologists.

### Cell Growth Assay

Cells were seeded into a 96-well plate at a density of 2×10^3^/well and were incubated in DMEM containing 5% FBS at 37°C in a 5% CO_2_ incubator for 5 days. Cell viabilities were studied using the CCK-8 assay according to the manufacturer’s protocols (Dojindo, Kumamoto, Japan). Briefly, 90 µl of serum-free culture medium and 10 µl of CCK-8 solutions were added to each sample. After incubation for 2 h, absorbance at 450 nm was measured with an enzyme-linked immunosorbent assay analyzer. Six parallel replicates were read for each sample.

## Results

### E2 Induces δEF1 Expression in an ER-α-dependent Manner

**Figure 1:**
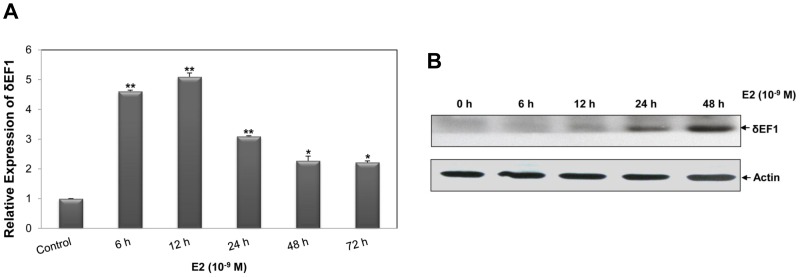
E2 treatment up-regulates δEF1 expression in MCF-7 cells. **A.** MCF-7 cells were treated with 10^−9^ M E2 at the indicated time points. The up-regulation of δEF1 mRNA was verified by Q-PCR. GAPDH was used to normalize the δEF1 level. * indicates p<0.05 in unpaired Student’s t-test compared with the control. ** indicates p<0.01 in unpaired Student’s t-test compared with control. The data represent three independent experiments. **B.** MCF-7 cells were treated with 10^−9^ M E2 at the indicated time points. The expression of δEF1 protein was verified by Western Blot. Actin was used to normalize the δEF1 level.

To assess the possible role of δEF1 in the estrogen cascade, MCF-7 cells were treated with 10^−9^ M E2 at the indicated time points. Q-PCR analysis revealed that E2 significantly up-regulated the expression of δEF1 at the mRNA level (Fig. 1A). The δEF1 mRNA increased 4.5–5 folds at as early as 6–12 h after E2 treatment. In 24–72 h, the magnitude of the δEF1 induction was maintained at the level of 2.2–3-folds above control. Accordingly, δEF1 protein was consistently increased through 48 h by E2 as demonstrated by Western Blot (Fig. 1B).

**Figure 2:**
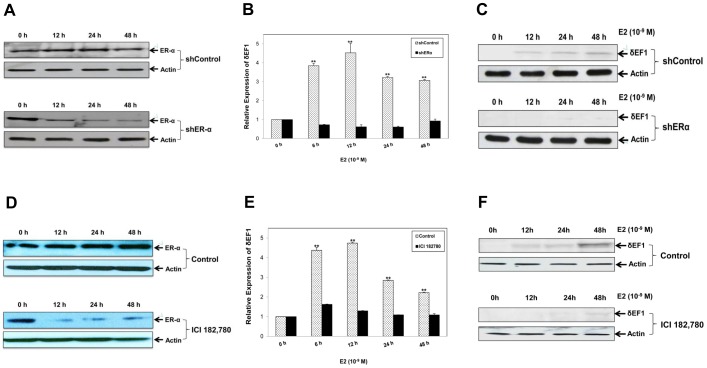
E2-induced up-regulation of δEF1 is ER-α-dependent. **A.** The ER-α-specific shRNA (shER-α) was transiently transfected into MCF-7 cells. Control cells (shControl) were treated with the scrambled shRNA. At the indicated time points, the expression of ER-α protein was verified by Western Blot. Actin was used to normalize the ER-α level. **B.** shER-α or shControl was transiently transfected into MCF-7 cells followed by treatment with 10^−9^ M E2. The expression of δEF1 mRNA was determined at the indicated time points after treatment using Q-PCR. GAPDH was used to normalize the δEF1 level. ** indicates p<0.01 in unpaired Student’s t-test compared with shControl. The data represent three independent experiments. **C.** shER-α or shControl was transiently transfected into MCF-7 cells followed by treatment with 10^−9^ M E2. The expression of δEF1 protein was determined at the indicated time points following treatment using Western Blot. Actin was used to normalize the δEF1 level. **D.** MCF-7 cells were pre-incubated with ICI 182,780 (1 µM) at the indicated time points. The expression of ER-α protein was verified by Western Blot. Actin was used to normalize the ER-α level. **E.** MCF-7 cells were pre-incubated with ICI 182,780 (1 µM) for 0.5 h followed by treatment with 10^−9^ M E2. The expression of δEF1 mRNA was determined at the indicated time points after treatment using Q-PCR. GAPDH was used to normalize the δEF1 level. ** indicates p<0.01 in unpaired Student’s t-test compared with control. The data represent three independent experiments. **F.** MCF-7 cells were pre-incubated with ICI 182,780 (1 µM) for 0.5 h followed by treatment with 10^−9^ M E2. The expression of δEF1 protein was determined at the indicated time points following treatment using Western Blot. Actin was used to normalize the δEF1 level.

Given that estrogen functions primarily via ER-α in MCF-7 cells [1], we envisioned that the E2-induced expression of δEF1 would be ER-dependent. MCF-7 cells were transiently transfected with a shRNA targeting ER-α in the presence or absence of E2. The knockdown of ER-α was assessed by Q-PCR and Western Blot (Fig. 2A). The results showed that the E2-induced up-regulation of δEF1 mRNA was completely abolished by the knockdown of ER-α (Fig. 2B). Western Blot analysis further confirmed that E2 treatment induced δEF1 expression at the protein level, whereas the knockdown of ER-α dramatically reduced this effect (Fig. 2C). Similarly, MCF-7 cells were treated with the ER antagonist ICI 182,780 in the presence of E2 [31]. As seen in Fig. 2D, down-regulation of ER-α expression by ICI 182,780 was observed. Q-PCR (Fig. 2E) and Western Blot (Fig. 2F) showed that the E2-induced up-regulation of δEF1 was significantly inhibited by the ER blockade using ICI 182,780. Taken together, these data demonstrate that E2 promotes δEF1 expression in an ER-α-dependent manner in MCF-7 cells.

### E2 Up-regulates δEF1 by Differentially Regulating PI3K and NF-κB Pathways

**Figure 3:**
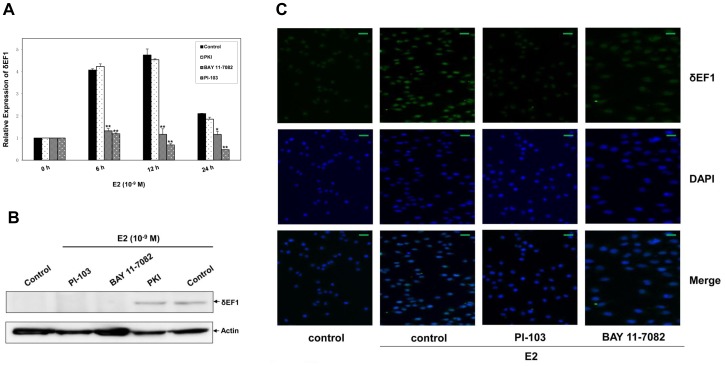
E2 treatment regulates δEF1 expression via PI3K and NF-κB pathways. **A.** MCF-7 cells were pre-incubated with PKI (5 µg/µl), BAY 11-7082 (10 µM), or PI-103 (10 µM) for 0.5 h followed by treatment with 10^−9^ M E2. The expression of δEF1 mRNA was determined at the indicated time points following treatment using Q-PCR. GAPDH was used to normalize the δEF1 level. * indicates p<0.05 in unpaired Student’s t-test compared with control. ** indicates p<0.01 in unpaired Student’s t-test compared with control. The data represent three independent experiments. **B.** MCF-7 cells were pre-incubated with PKI, BAY 11-7082, or PI-103 for 0.5 h followed by treatment with 10^−9^ M E2. The expression of δEF1 protein was determined at 24 h following treatment using Western Blot. Actin was used to normalize the δEF1 level. **C.** MCF-7 cells were pre-incubated with PKI, BAY 11-7082, or PI-103 for 0.5 h followed by treatment with 10^−9^ M E2. The expression of δEF1 protein was determined at 24 h following treatment using immunofluorescence. Scale bars, 50 µm.

The estrogen/ER cascade has been reported to potentially function through PI3K [32], PKA [33], and NF-κB [34] pathways in breast cancer. To identify the downstream signaling mechanism(s) of E2-induced δEF1 expression, MCF-7 cells were treated with the following inhibitors in the presence of E2: PI-103 (PI3K inhibitor), PKI (PKA inhibitor), and BAY 11-7082 (NF-κB inhibitor). As shown in Fig. 3A, the Q-PCR results revealed that treatment with PI-103 or BAY 11-7082 significantly blocked E2-induced δEF1 expression at the mRNA level when compared with the control. However, inhibition of PKA signaling by PKI does not interfere with the up-regulation of δEF1 mRNA by E2. Western Blot (Fig. 3B) and immunofluorescence (Fig. 3C) assays further confirmed that the inhibition of the PI3K or NF-κB signaling pathway abolished the stimulatory function of E2 on δEF1 at the protein level. Taken together, these observations suggest that the PI3K and NF-κB pathways contribute to E2-regulated δEF1 expression in MCF-7 cells.

### Ectopic Expression of δEF1 Down-regulates ER-α

**Figure 4:**
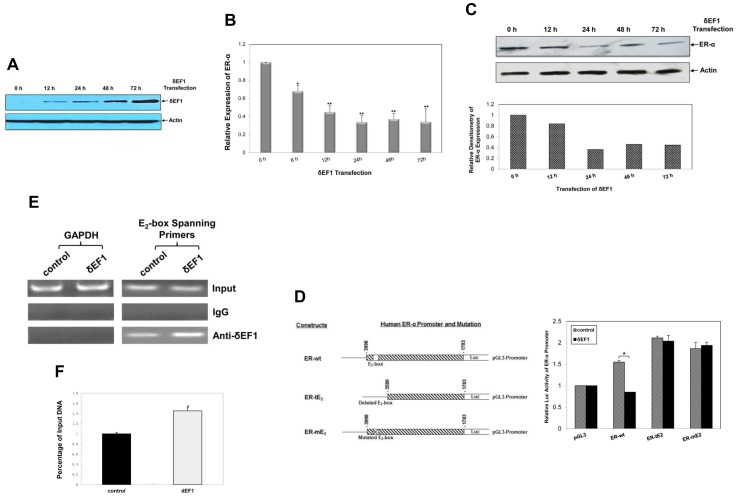
Ectopic expression of δEF1 represses ER-α transcription by binding to E_2_-box on its promoter. **A.** The δEF1 expression plasmid was transiently transfected into MCF-7 cells. At the indicated time points following transfection, the expression of δEF1 protein was verified by Western Blot. Actin was used to normalize the ER-α level. **B.** MCF-7 cells were transiently transfected with the δEF1 expression plasmid. At the indicated time points following transfection, the down-regulation of ER-α mRNA was verified by Q-PCR. GAPDH was used to normalize the ER-α level. * indicates p<0.05 in unpaired Student’s t-test compared with control. ** indicates p<0.01 in unpaired Student’s t-test compared with control. The data represent three independent experiments. **C.** MCF-7 cells were transiently transfected with the δEF1 expression plasmid. At the indicated time points following transfection, the expression of ER-α protein was examined by Western Blot. Actin was used to normalize the ER-α level. **D.** Sequential deletion (ER-tE_2_) and mutation (ER-mE_2_) of E_2_-box on the human ER-α promoter were fused to the luciferase reporter. MCF-7 cells on 24-well plates were co-transfected with the δEF1 expression plasmid (1 µg/well) or wild-type (ER-wt), truncated (ER-tE_2_), or mutated (ER-mE_2_) ER-α promoter luciferase reporter constructs (1 µg/well). The luciferase activities of the extracts were determined at 24 h following transfection using a Betascope analyzer. The luciferase values are normalized with constitutive Renilla activities. * indicates p<0.05 in unpaired Student’s t-test compared with control. The data represent three independent experiments. **E.** The association of δEF1 with the ER-α promoter was assessed by ChIP analysis in MCF-7 cells using a polyclonal antibody against δEF1 or an unrelated IgG antibody. The amplified ER-α promoter fragment of the E_2_-box-containing sequence is shown. The amount of DNA in the input lane confirms the equal loading of chromatin. **F.** δEF1 transfection significantly enhanced its recruitment to the endogenous ER-α promoter by quantitative ChIP analysis. * indicates p<0.05 in unpaired Student’s t-test compared with vector alone. The data represent three independent experiments.

Since δEF1 expression was correlated with the ER presence of breast cancer in our previous study [25], we speculated that the up-regulation of δEF1 would affect ER-α expression. MCF-7 cells were transiently transfected with the δEF1 expression plasmid. The overexpression of δEF1 was confirmed by Western Blot (Fig. 4A). As shown in Fig. 4B, a significant down-regulation of ER-α mRNA was observed following the overexpression of δEF1. The transfection of δEF1 for 24 h resulted in a maximal 67% decrease in the expression of ER-α compared with that of the control. Moreover, Western Blot confirmed the δEF1-induced inhibition of ER-α expression at the protein level (Fig. 4C). Using densitometry to quantify, δEF1 overexpression for 24–72 h resulted in a maximal 55–65% decrease in the expression of ER-α protein level compared with the control.

We next assessed whether δEF1 is a true repressor of ER-α transcription using reporter gene assays [35]. As shown in Fig. 4D, δEF1 significantly repressed the human ER-α promoter activity of the wild-type -3998/−1783 reporter by approximately 45% relative to the control without δEF1 transfection. Furthermore, we found that δEF1 inhibited the promoter activity of ER-α in a dose-dependent manner (Fig. S1). Given that δEF1 functions as a transcriptional repressor by binding to the E_2_-boxes [CA(C/G)(C/G)TG] in the promoter region of target genes [9], [26], we performed a search using the transcription factor database TESS and identified an E_2_-box (CACCTG) at the position -3669/−3663 of the ER-α promoter. A truncated ER-α promoter-reporter construct was thus generated as ER-tE_2_ for testing (Fig. 4D). The results showed that the deletion of the E_2_-box completely abolishes the δEF1-induced transrepression of ER-tE_2_ compared with the control without δEF1 transfection. Furthermore, we made a mutation on the E_2_-box (CACCTG to CATCTG) using site-directed mutation experiment. We found that the mutation of the E_2_-box is sufficient to interfere with the δEF1-inhibited transcription of the ER-α promoter (Fig. 4D). Importantly, the ChIP assays indicated that δEF1 is able to bind to the ER-α promoter during basal conditions in an E_2_-box-dependent manner (Fig. 4E). The overexpression of δEF1 resulted in a 1.5-fold increase in its binding to the endogenous ER-α promoter (Fig. 4F), suggesting that overexpressed δEF1 inhibits ER-α transcription in MCF-7 cells by a direct binding to the ER-α promoter.

### Expression of δEF1 and ER-α is Inversely Correlated in Breast Cancer

**Figure 5:**
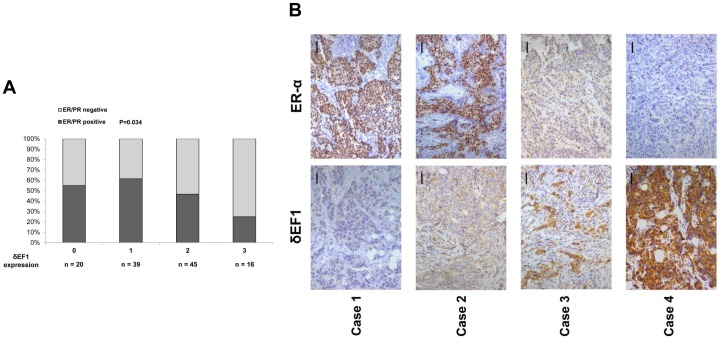
Expression of δEF1 and ER-α is inversely correlated in breast cancer specimens. **A.** The positive percentage analysis for ER-α indicates a negative correlation with δEF1 expression in breast cancer tumors from 120 subjects. **B.** Representative images of immunohistochemical staining of δEF1 and ER-α in two serial sections of the same tumor from four cases are shown. Scale bars, 50 µm.

To better understand the correlation of δEF1 and ER-α in breast cancer, we collected breast cancer tissue samples from 120 human subjects. We segregated the samples into four groups based on their δEF1 expression levels. The expression of ER-α in each group was represented by the numbers or percentages of positive cases. The results showed that the increased expression (positive percentage) of ER-α was negatively correlated with δEF1 expression (Fig. 5A and Table 1). Moreover, immunohistochemical staining of four representative subjects confirmed the inverse relationship between δEF1 and ER-α expression (Fig. 5B), which is consistent with our findings that δEF1 down-regulates ER-α in breast cancer.

**Table 1:** Inverse correlation between δEF1 and ER-α expression in breast cancers.

δEF1 staining intensity scores	No. of IDC-NOS specimens	
	ER-α/PR(+)	ER-α/PR(−)
0	11	9
1	24	15
2	21	24
3	4	12

*p*  = 0.034.

*p* values were calculated by Spearman’s Rank-Correlation test (n  = 120).

### δEF1 Confers Tamoxifen Resistance by Altering ER-α Expression

**Figure 6:**
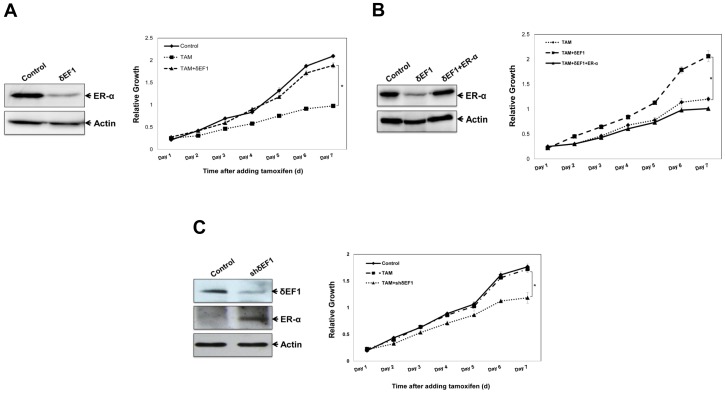
Ectopic expression of δEF1 decreases sensitivity of breast cancer cells to tamoxifen. **A.** MCF-7 cells were stably transfected with the δEF1 expression plasmid. The expression of the ER-α protein was determined using Western Blot. Actin was used to normalize the ER-α level. MCF-7 cells stably transfected with δEF1 were treated with 10^−6^ M tamoxifen. At the indicated time points, cell growth was measured using the CCK-8 assay. * indicates p<0.05 in unpaired Student’s t-test compared with the control. **B.** ER-α expression plasmid was introduced into MCF-7 cells that were stably transfected with δEF1 followed by treatment with tamoxifen (10^−6^ M). The ER-α protein expression was determined using Western Blot. Actin was used to normalize the ER-α level. At the indicated time points, the cell growth was assessed using the CCK-8 assay. * indicates p<0.05 in unpaired Student’s t-test compared with control. **C.** MDA-MB-231 cells were stably transfected with shRNA targeting δEF1. The expressions of δEF1 and ER-α protein were determined using Western Blot. Actin was used to normalize the ER-α level. MDA-MB-231 cells stably transfected with shδEF1 were treated with 10^−6^ M tamoxifen. At the indicated time points, cell growth was measured using the CCK-8 assay. * indicates p<0.05 in unpaired Student’s t-test compared with the control.

Given that the loss of or reduced ER-α expression is a primary mechanism for tamoxifen resistance, we next tested whether δEF1 overexpression in breast cancer cells would confer resistance to tamoxifen-mediated cell growth inhibition and cell death. We stably transfected MCF-7 cells with δEF1, which were subsequently treated with tamoxifen, and measured cell growth under different conditions. Our results showed that the growth rate of MCF-7 cells was reduced by the tamoxifen treatment compared with the control treatment. The overexpression of δEF1 prevented the tamoxifen-induced inhibition of cell growth (Fig. 6A). Importantly, the re-expression of ER-α reduced the effects of δEF1 on the sensitivity of the cells to tamoxifen (Fig. 6B). Moreover, δEF1-transfected MCF-7 cells were treated with fulvestrant (also termed as ICI 182,780), which is a selective ER down-regulator. As shown in Fig. S2, fulvestrant treatment exhibited results comparable to those of tamoxifen.

We previously showed that the ER-negative breast cancer cells MDA-MB-231 expressed high levels of endogenous δEF1 [25]. We therefore investigated whether the knockdown of δEF1 using RNA interference may lead to an increased expression of ER-α and tamoxifen sensitivity. A control- or δEF1-targeted shRNA was introduced into MDA-MB-231 cells followed by treatment with tamoxifen. The knockdown of δEF1 expression was confirmed by Western Blot and was accompanied by a significantly increased ER-α expression (Figure 6C). The growth rates of the shδEF1-treated MDA-MB-231 cells were significantly reduced by the addition of tamoxifen compared with the control cells (Figure 6C). The data verified that depletion of δEF1 in MDA-MB-231 cells allowed restoration of the cell sensitivity to tamoxifen. Taken together, we have found an important mechanism that confers the resistance of breast cancer cells to tamoxifen treatment. δEF1 does it through down-regulating ER-α expression.

## Discussion

Resistance to tamoxifen therapy represents a major barrier to the successful treatment of breast cancer, and ER-α expression is currently the main biomarker of response to tamoxifen treatment. Elucidating the regulation of ER-α expression may reveal new therapeutic targets for overcoming tamoxifen resistance. In the present study, we showed that ectopic expression of δEF1 resulted in a loss of sensitivity to tamoxifen in MCF-7 cells, which is mediated by a reduction of ER-α. In breast cancer specimens, we confirmed that tumors with high levels of δEF1 exhibited dramatically reduced ER-α expression. We have identified a mechanism by which δEF1 confers the development of resistance to tamoxifen in breast cancer by lowering ER-α expression.

Tamoxifen resistance is attributed to a number of cellular changes, among which the loss of ER-α expression/function is implicated as a key phenotype. Indeed, ER-negative breast cancers rarely respond to tamoxifen treatment. Accordingly, the levels of ER-α protein in the tumor correlates with their sensitivities to tamoxifen therapy [3], [36]. In this study, we found that δEF1 overexpression repressed ER-α expression in MCF-7 cells, recapitulates a loss of response to tamoxifen treatment in vitro. Using luciferase and ChIP assays, we further demonstrated that δEF1 is recruited to the E_2_-box of the ER-α promoter, where it binds and inhibits transcription. On the other hand, a deletion or mutation of the E_2_-box abolished the inhibitory effect of δEF1 on the ER-α promoter, an effect consistent with the previous finding that δEF1 acts as a transcriptional repressor by binding to the E_2_-boxes of target genes in multiple cell lineages [9], [10], [13], [25], [26]. In breast cancer specimens, we confirmed that the level of ER-α was negatively correlated with the level of δEF1 expression, which supports well our published data that expression of δEF1 was elevated in the breast cancer [25]. It is therefore plausible to infer that the up-regulation of δEF1 caused a reduced ER-α expression in breast cancers and may have contributed to the resistance of this subset of breast cancers to tamoxifen therapy. It is also worth pointing out that in the tumors with high δEF1 expression, even if they are ER-α-positive, the level could be very low that they may respond poorly to tamoxifen treatment.

Our continued study in MDA-MD-231 cells was further supportive. MDA-MD-231 cells were known to be inherently ER-negative and were unresponsive to tamoxifen. The knockdown of δEF1 in these cells resulted in a re-activation of ER-α expression and rendered the cells sensitive to the inhibitory effect of tamoxifen on growth. A number of causes have been identified that could contribute to the loss of ER-α expression, such as homozygous deletion, loss of heterozygosity, or *ER* gene mutation [37]. Increasing evidence has also started to point towards epigenetic alterations which may play a role in the inactivation of ER expression. For example, *Papidus et al.* reported that the *ER* CpG island is unmethylated in normal breast tissue and most ER-positive tumor cell lines, whereas it is methylated in 50% of unselected primary breast cancers and most ER-negative breast cancer cell lines, including MDA-MB-231. The methylation of these CpG cluster sites is associated with either reduced or absent ER expression [38]. Moreover, treatment with DNA methyltransferase (DNMT) and/or histone deacetylase (HDAC) inhibitors induce re-expression of ER-α mRNA and protein in MDA-MB-231 cells, which consequently restores the cell response to estrogen [39]. A recent report also demonstrated that Twist, which is another member of the zinc finger-homeodomain transcription factor family, contributes to the hormone resistance in breast cancer by down-regulating ER-α. Mechanistically, Twist interacts with DNMT3B and HDAC1 at the ER promoter, causing histone deacetylation and promoter hyper-methylation, further reducing ER transcription levels [40]. In this study, we presented the evidence that δEF1 is a novel regulation mechanism which mediates the loss of ER activity observed in breast cancer, and may contribute to the generation of hormone-resistant breast cancer.

δEF1 is increasingly recognized as a critical molecule that regulates gene transcription and cell activities. Recent reports have indicated that δEF1 acts as a master regulator of malignant breast cancer progression by differentially regulating a series of downstream factors. For example, δEF1 primarily functions at the crossroad between cellular proliferation and differentiation by targeting p21 and E-cadherin pathways [19], [25]. δEF1 was also shown to induce MMP-1 expression in MDA-MB-231 breast cancer cells, resulting in osteolytic bone metastasis [18]. Meanwhile, a few important molecules have been identified that regulate δEF1 expression in mammary tissues. The TGF-β family members, including TGF-β and BMP-6, are reported to alter δEF1 levels and exert their function in the regulation of breast cancer progression and metastasis [19], [25], [41]. We now report that E2 treatment up-regulates δEF1 expression in MCF-7 cells in an ER-dependent manner involving PI3K and NF-κB pathways. Consistent with our research, Park *et al.* reported that E2 induces the metastatic potential of ovarian cancer by up-regulating Snail and Slug, which are also members of the zinc finger-homeodomain transcription factor family [42].

In this study, we provide a novel finding for a potential mechanism of the estrogen/δEF1-mediated endocrine resistance of breast cancer. The effect is mediated through the down-regulation of ER-α. We therefore suggest that δEF1 or pathways downstream to δEF1 may be viable therapeutic targets. Inhibition of δEF1 expression to restore ER-α level will represent a new therapeutic strategy for overcoming endocrine resistance in breast cancers. Further investigation *in vivo* will be necessary to validate the targets and the pharmacological benefits.

## Supporting Information

Figure S1 **Luciferase assay showing repression of ER-α promoter by δEF1 in a dose-dependent manner.**
(DOC)

Figure S2 **Cell growth assay showing loss of sensitivity to fulvestrant in δEF1-transfected MCF-7 cells.**
(DOC)
