# Supplementary material for: δEF1 Down-Regulates ER-α Expression and Confers Tamoxifen Resistance in Breast Cancer
Source: PLoS One. 2012 Dec 21;7(12):e52380. doi: 10.1371/journal.pone.0052380 (PMC3528679; doi:10.1371/journal.pone.0052380)
Supplement: Figure S1 — Luciferase assay showing repression of ER-α promoter by δEF1 in a dose-dependent manner. (DOC) [file pone.0052380.s001.doc]

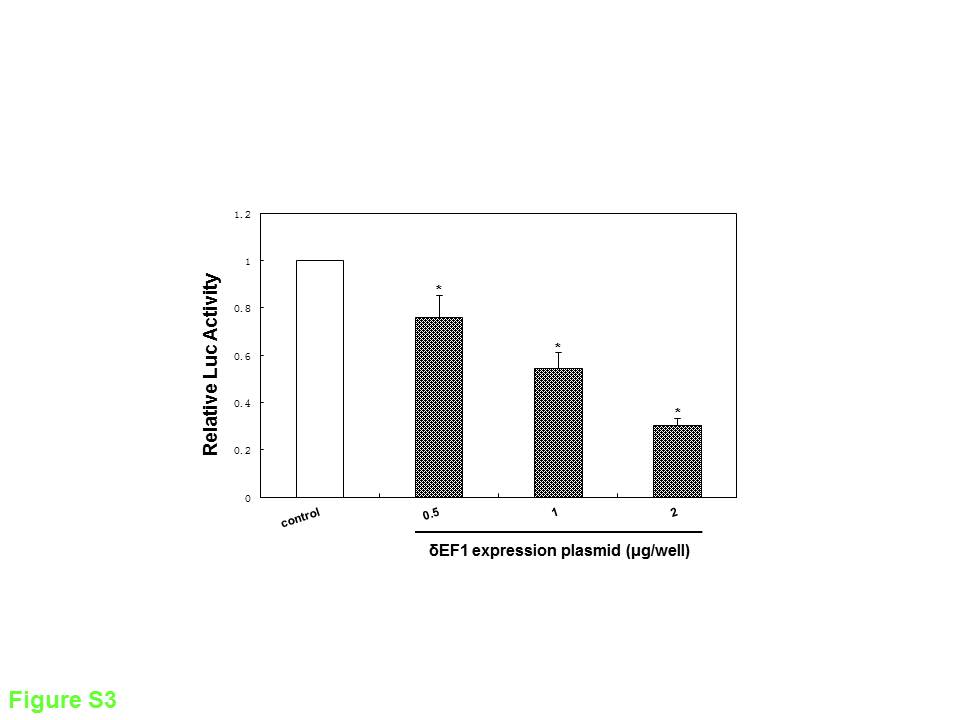


**Figure S1.** δEF1 represses the transcriptional activity of ER-α in a dose-dependent manner. MCF-7 cells were co-transfected with different amount of δEF1 expression plasmid and ER-wt promoter reporter. The luciferase activity is determined after 24 h of transfection, using a Betascope analyzer. Luciferase values are normalized with Renilla activities. * indicates p<0.05 in unpaired student t test when compared with the vector alone. Data represent three independent experiments.
