# Supplementary material for: δEF1 Down-Regulates ER-α Expression and Confers Tamoxifen Resistance in Breast Cancer
Source: PLoS One. 2012 Dec 21;7(12):e52380. doi: 10.1371/journal.pone.0052380 (PMC3528679; doi:10.1371/journal.pone.0052380)
Supplement: Figure S2 — Cell growth assay showing loss of sensitivity to fulvestrant in δEF1-transfected MCF-7 cells. (DOC) [file pone.0052380.s002.doc]

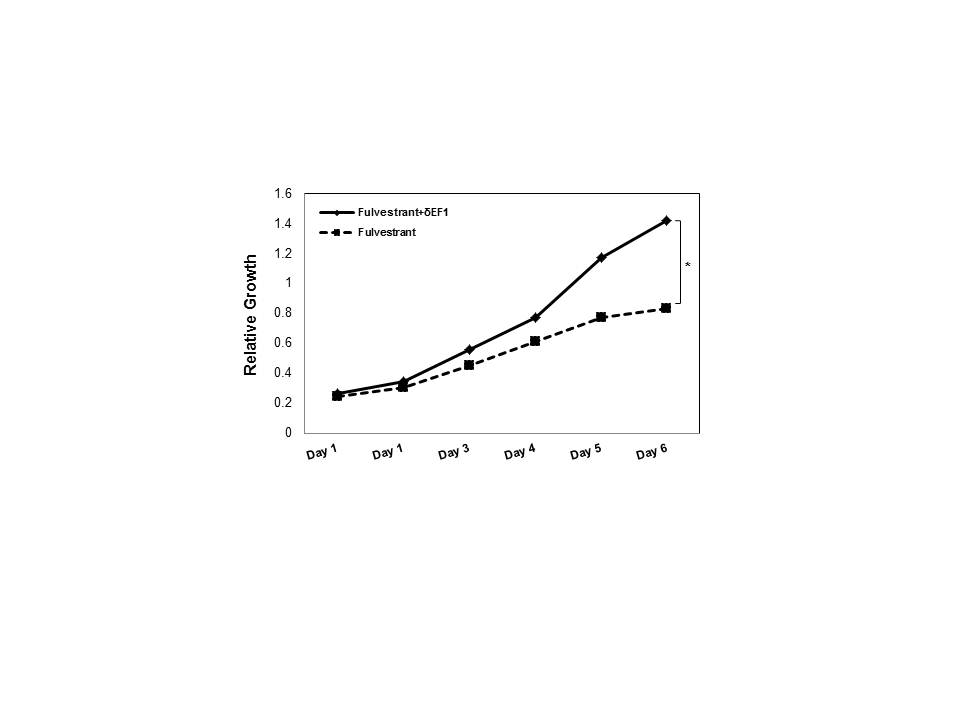


**Figure S2.** Ectopic expression of δEF1 decreases sensitivity of breast cancer cells to fulvestrant. MCF-7 cells were stably transfected with the δEF1 expression plasmid or empty vector control, followed by the treatment with 10^-9^ M fulvestrant. At the indicated time points, cell growth was measured using the CCK-8 assay. * indicates p<0.05 in unpaired Student’s t-test compared with controls.
